# Troponin Testing for Assessing Sepsis-Induced Myocardial Dysfunction in Patients with Septic Shock

**DOI:** 10.3390/jcm8020239

**Published:** 2019-02-12

**Authors:** June-Sung Kim, Muyeol Kim, Youn-Jung Kim, Seung Mok Ryoo, Chang Hwan Sohn, Shin Ahn, Won Young Kim

**Affiliations:** Department of Emergency Medicine, University of Ulsan College of Medicine, Asan Medical Center, Seoul 05505, Korea; jsmeet09@gmail.com (J.-S.K.); kurt0217@naver.com (M.K.); yjkim.em@gmail.com (Y.-J.K.); chrisryoo@naver.com (S.M.R.); schwan97@gmail.com (C.H.S.); ans1023@gmail.com (S.A.)

**Keywords:** septic shock, cardiomyopathy, troponin, biomarker

## Abstract

(1) Background: Myocardial dysfunction in patients with sepsis is not an uncommon phenomenon, yet reported results are conflicting and there is no objective definition. Measurement of troponin may reflect the state of the heart and may correlate with echocardiographically derived data. This study aimed to evaluate the role of admission and peak troponin-I testing for the identification of sepsis-induced myocardial dysfunction (SIMD) by transthoracic echocardiography (TTE). (2) Methods: This was a retrospective cohort study using a prospective registry of septic shock at an Emergency Department from January 2011 and April 2017. All 1,776 consecutive adult septic shock patients treated with protocol-driven resuscitation bundle therapy and tested troponin-I were enrolled. SIMD was defined as left ventricular (LV) systolic/diastolic dysfunction, right ventricular (RV) diastolic dysfunction, or global/regional wall motion abnormalities (WMA). (3) Results: Of 660 (38.4%) septic shock patients with an elevated hs-TnI (≥0.04 ng/mL) at admission, 397 patients underwent TTE and 258 cases (65%) showed SIMD (LV systolic dysfunction (*n* = 163, 63.2%), LV diastolic dysfunction (*n* = 104, 40.3%), RV dysfunction (*n* = 97, 37.6%), and WMA (*n* = 186, 72.1%)). In multivariate analysis, peak hs-TnI (odds ratio 1.03, 95% confidence interval 1.01–1.06, *p* = 0.008) and ST-T wave changes in the electrocardiogram (odds ratio 1.82, 95% confidence interval 1.04–2.39, *p* = 0.013) were associated with SIMD, in contrast to hs-TnI level at admission. The area under the curve of peak hs-TnI was 0.668. When the peak hs-TnI cutoff value was 0.634 ng/mL, the sensitivity and specificity for SIMD were 58.6% and 59.1%, respectively. 4) Conclusions: About two-thirds of patients with an elevated hs-TnI level have various cardiac dysfunctions in terms of TTE. Rather than the initial level, the peak hs-TnI and ST-T change may be considered as a risk factor of SIMD.

## 1. Introduction

Septic shock is still a leading cause of death worldwide as it can induce multi-organ failure [1,2]. Cardiac dysfunction, referred to as sepsis-induced myocardial dysfunction (SIMD), is a loosely defined syndrome and presents in various ways, such as myocardial injury with cardiac biomarker elevation, myocardial dysfunction on echocardiography, and hemodynamic instability [3]. SIMD is a common complication (40–60%), which could possibly be the result of increased circulating catecholamine and cytokine levels in severe sepsis and septic shock and its presence significantly worsens the outcome [4,5]. SIMD may involve either the left ventricle (LV), the right ventricle (RV) or both. This may manifest as systolic dysfunction, LV diastolic dysfunction, RV dysfunction, global hypokinesia or regional wall motion abnormalities [6]. Early recognition, aggressive supportive therapy, and appropriate catecholamine regimen are mandatory in order to improve survival. Although echocardiography has been as a golden tool for SIMD, the echocardiographic findings of SIMD are still poorly defined, and it is not practical for performing echocardiography for every sepsis patient during early resuscitation due to its cost and limited round-the-clock availability.

Troponin-I is the subunit of the troponin complex which is exclusively of cardiac origin. It is sensitive and a specific biomarker of myocardial damage and is related to prognosis in non-acute coronary syndrome such as pulmonary embolism, trauma, stroke, and subarachnoid hemorrhage [7]. Recent studies have demonstrated that cardiac troponin release in sepsis is associated with poor outcomes, including higher mortality and longer length of stay in an intensive care unit [8]. Theories for troponin elevations in septic shock include inflammation, increased myocardial wall stress by volume overload, toxicity by medications, and kidney dysfunction [9]. However, the results of the studies investigating the impact of troponin levels for SIMD in sepsis are not concordant. Although some research showed a significant association between troponin and SIMD on echocardiography such as LV systolic, diastolic dysfunction or RV dysfunction, [10,11] others did not [12]. In this regard, there is limited data regarding the relationship between troponin elevation and different types of SIMD [13,14,15].

We hypothesized that measurement of troponin on admission may provide information about the state of heart and may be complementary to echocardiographically derived data. Moreover, we postulated that the degree of increase in troponin level on serial measurement would be of incremental value in risk stratification. To test this hypothesis, we used a prospective registry of septic shock to evaluate the role of admission and peak troponin-I testing for the identification of SIMD by transthoracic echocardiography (TTE) in septic shock patients.

## 2. Materials and Methods

### 2.1. Setting

A retrospective, observational, single-center, registry-based study was performed at the emergency department (ED) at a tertiary referral academic center in Seoul, Korea, with 2800 beds and 120,000 ED patients treated annually. The study period was from 1 January 2011 to 31 April 2017, and all of the consecutive adult patients (≥18 years) analyzed had been enrolled in the septic shock registry [16]. This study was approved by the Institutional Review Board of Asian Medical Center (Study No. 2016-0548), which waived the required for informed consent because of the retrospective nature of the analysis.

### 2.2. Patients and Definition of Sepsis

Because this study was conducted prior to the recent announcement of new sepsis definitions, septic shock was defined in our patient cohort as refractory hypotension (mean arterial pressure ≤ 70 mmHg), or a blood lactate level of at least 4 mmol/L despite an adequate volume resuscitation [17]. Exclusion criteria in this cohort included an age below 18 years, pregnancy, a “do not resuscitate” order, a transfer to another hospital without adequate treatment, or a transfer from another hospital after recovering from a shock state. We also excluded any patients without a high-sensitivity troponin-I (hs-TnI) level or TTE check during their hospital stay and who showed abnormalities in past echocardiographic parameters such as LV dysfunction, RV dysfunction, or wall motion abnormalities (WMA) induced by previous assumed ischemic insults.

### 2.3. Clinical and Laboratory Data

After finalizing the patients to be included in our present analysis from the enrolled registry, we collected clinical, laboratory, and radiological data from their electronic medical records including age, sex, past medical history, chief complaints, and vital signs. A 12-lead electrocardiogram (EKG) had been conducted in all cases and was used to interpret whether ST segment and T wave changes (STTC) were present. STTC included ST-segment elevation (≥2 mm in V2, 3, and ≥1 mm in others), ST-segment depression (≥0.5 mm) and T-wave inversion (≥2 mm) in two consecutive leads. Blood samples were obtained from all registry enrolled patients within 15 minutes after their visit to the ED. White blood cell counts, hemoglobin, C-reactive protein, blood urine nitrogen, creatinine, coagulation factors, and liver function tests were reported. In addition, Sequential Organ Failure Assessment (SOFA) scores were calculated for each patient based on their initial admission data.

### 2.4. High-Sensitivity Troponin I Assay

The serum hs-TnI levels had been checked in all of the septic shock patients we included in this current study and were repeated at least once a day during the hospital stay. The initial and peak level during admission was recorded in each case. Measurements of the hs-TnI were conducted with a three-site sandwich immunoassay using TnI-Ultra direct chemiluminometric technology (ADVI Centaur XPT; Siemens, Munich, Germany). A value of 0.04 ng/mL, which represents the 99^th^ percentile reported in the normal population, was considered to be cut-off value [18].

### 2.5. Echocardiographic Variables

**Figure 1:**
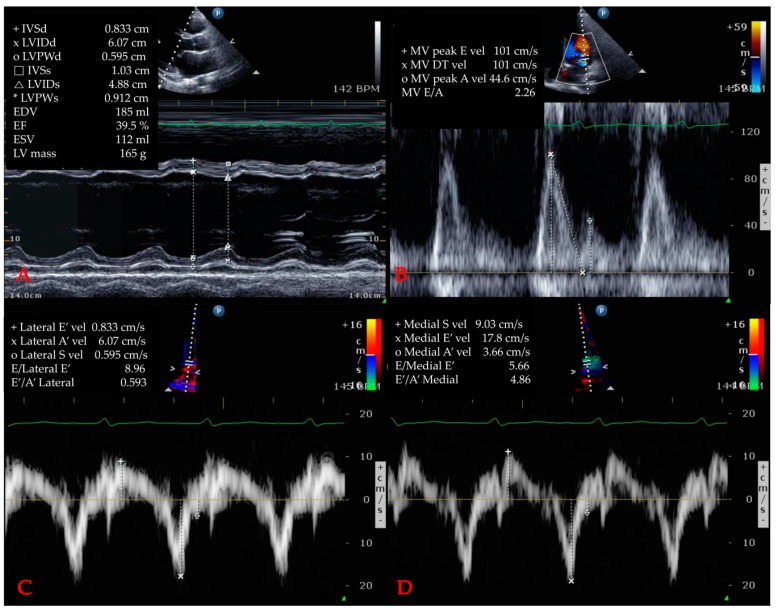
Example of echocardiography. (**A**) left ventricle ejection fraction measurement via Teichholz method. (**B**) measurement of mitral valve parameters. (**C**) measurement of E/e’ via tissue Doppler image (lateral). (**D**) measurement of E/e’ via tissue Doppler image (medial) Abbreviations: IVSd = interventricular septum (diastolic); LV = left ventricle; LVIDd = left ventricular internal diameter (diastolic); LVPWd = left ventricle posterior wall (diastolic); IVSs = interventricular septum (systolic); LVIDs = left ventricular internal diameter (systolic); LVPWs = left ventricle posterior wall (systolic); EDV = end-diastolic volume; EF = ejection fraction; ESV = end-systolic volume; MV = mitral valve; E = peak early diastolic transmitral flow; vel = velocity; DT = deceleration time; A = peak late diastolic transmitral flow velocity; E’ = peak early diastolic mitral annulus velocity; A’ = peak late diastolic mitral annulus velocity.

Cardiac dysfunction induced by SIMD was diagnosed via two-dimensional TTE and was defined as any evidence of LV systolic/diastolic dysfunction, RV dysfunction, or WMA [19]. TTE was usually performed for patients with an elevated hs-TnI by experienced cardiologists and/or sonographers after admission. Parasternal long and short axis views, apical 4 chamber and 2 chamber views were obtained in accordance with the guidelines of the American Society of Echocardiography. Via M-mode at parasternal long axis view, baseline echocardiographic parameters such as systolic/diastolic interventricular septum diameter, left ventricular internal diameter, posterior wall diameter, end-diastolic/systolic volume, peak early/late diastolic transmitral flow velocity, peak early/late diastolic mitral annulus velocity were measured. LV dysfunction included both systolic and diastolic abnormalities. The ejection fraction (EF) was measured using a modified Simpson method or Teichholz method. An EF below 50% was defined as LV systolic dysfunction as previously described [14]. Through tissue Doppler imaging at both the septal and lateral mitral origins on a four-chamber view, (e’) was obtained, and the peak mitral inflow (E) was gained on a pulsed-wave Doppler. E/e’ ratios above 15 were considered to indicate diastolic dysfunction. In addition, RV dysfunction was defined as dilation or a decreased EF. WMA was defined as global hypokinesia or regional wall motion abnormalities extending beyond the geographic territory of a single epicardial artery. Figure 1 shows an example of echocardiography in this study. The primary objective of these measurements was to correlate initial and peak hs-TnI levels during admission with cardiac dysfunctions on echocardiography. 

### 2.6. Statistics

Continuous variables were expressed as the median with the interquartile range. Categorical data were analyzed using the chi-square test or with the Fisher’s exact test. Normality of distribution was examined with the Kolmogorov-Smirnov test. The Mann-Whitney U test was used for comparisons between two groups with and without outcomes, i.e., SIMD (+) vs. SIMD (−). Variables with an entry-level significance of *p* < 0.10 in the univariate analysis were included in a subsequent stepwise multivariate analysis, and the results were reported as odds ratios (OR) and 95% confidence intervals (CI). Receiver operating characteristic (ROC) curves and calculations of the area under the curve (AUC) were performed to compare the diagnostic ability of different variables. Sensitivity and specificity were calculated by standard statistical methods. The optimal cutoff value of hs-TnI for SIMD was determined with the Youden index (sensitivity + specificity-1) from ROC analysis. A *p*-value below 0.05 was considered significant. All statistical analyses were conducted using SPSS Statistics for Windows, version 23.0 (IBM Corp., Armonk, NY, USA).

## 3. Results

### 3.1. Study Population

**Figure 2:**
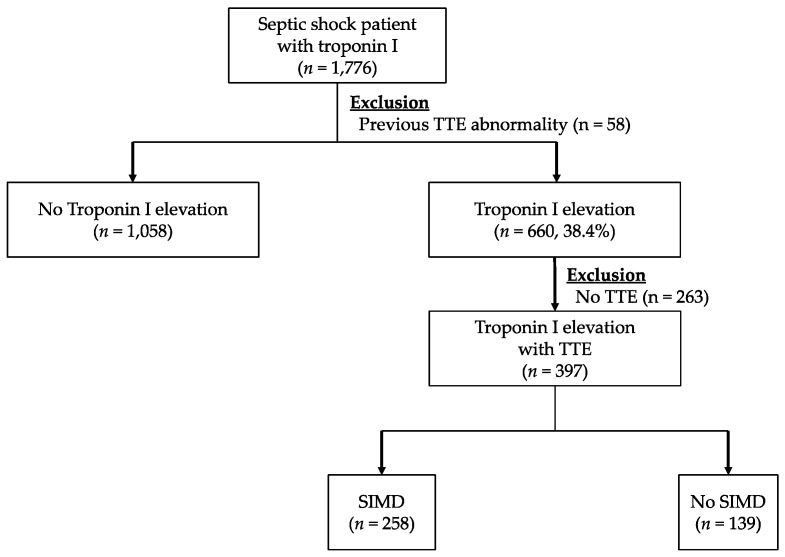
Flow diagram for the inclusion of patients with troponin I and a diagnosis of sepsis-induced myocardial dysfunction based on echocardiography. Abbreviations: TTE = transthoracic echocardiography, SIMD = sepsis-induced myocardial dysfunction.

Figure 2 shows the flowchart of the included study population. During the defined study period, 1,776 adult patients were enrolled in the septic shock registry at our hospital and 660 patients (38.4%) showed hs-TnI elevation on admission. After excluding 263 cases who did not undergo TTE, 397 patients were included in the final cohort for analysis, and 258 of these cases (64.9%) showed some form of cardiac dysfunctions in terms of TTE.

### 3.2. Baseline Characteristics and Frequency of Cardiac Dysfunction

The baseline characteristics of the study population are indicated in Table 1. The median age of the total cohort was 67.0 (range, 18.0–98.0) with slightly more men in both the SIMD (+) and (−) groups. Both hypertension and malignancy were common and, except for coronary artery disease (CAD), there were no statistically significant differences between these groups. A history of CAD was more common in the SIMD (+) group (17.8% vs. 10.1%; *p* = 0.041). Moreover, an EKG STTC finding was predominant in the SIMD (+) group (56.2% vs. 39.6%; *p* = 0.006). The initial vital signs and SOFA score were similar in both groups. In addition, the initial serum creatinine and lactate levels were not significantly different between the groups. The frequencies of various type of cardiac dysfunction are indicated in Table 2. LV systolic dysfunction was evident in 163 patients (63.2%), LV diastolic dysfunction in 104 (40.3%), RV dysfunction in 97 (37.6%), and WMA in 186 (72.1%). Overlap of three dysfunctions is presented with a Venn diagram in Table 2. Table 3 shows echocardiographic characteristics of cohorts. LV internal diameter, left atrium, LV posterior wall, end-systolic volume, end-diastolic volume, interventricular septum, and E/e’ were significantly higher in the SIMD (+) group, whereas LVEF and deceleration time were lower in SIMD (+).

### 3.3. Relationship Between Troponin-I and TTE Variables

**Figure 3:**
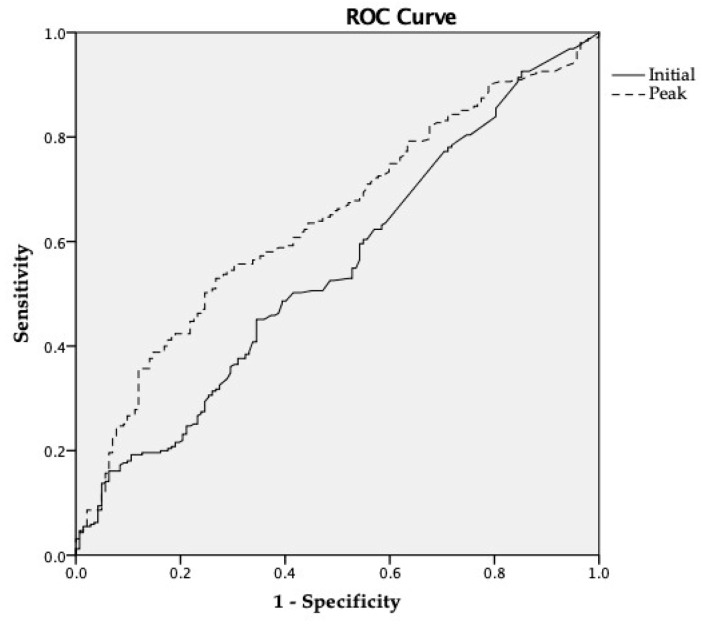
ROC curve of hs-TnI for diagnosis SIMD. Abbreviations: ROC = receiver operating characteristic; hs-TnI = high-sensitivity troponin-I; SIMD = sepsis-induced myocardial dysfunction.

The initial and peak troponin-I levels were calculated according to the different types of cardiac dysfunction (Table 4). The peak hs-TnI level was statistically different between the SIMD (+) and SIMD (−) groups except in patients with LV diastolic dysfunction. Moreover, the peak hs-TnI level was statistically higher in the patients with LV systolic dysfunction (1.609 vs. 0.540 ng/mL; *p* < 0.001), RV dysfunction (2.478 vs. 0.546 ng/mL; *p* < 0.001), and WMA (1.950 vs. 0.455 ng/mL; *p* < 0.001). The initial hs-TnI level was significantly higher in the LV diastolic dysfunction cases (0.160 vs. 0.085 ng/mL; *p* = 0.019). Through the ROC curve (Figure 3), we calculated the optimal cutoff values 0.668 ng/mL for peak hs-TnI and stratified the SIMD (+) group (AUC 0.634, sensitivity 58.6%, specificity 59.1%). Among the clinical and laboratory variables we tested, we found by multivariate analysis that EKG changes (OR 1.822; 95% CI 1.210–2.744; *p* = 0.004), and the peak hs-TnI level (OR 1.033; 95% CI 1.009–1.057; *p* = 0.008) were independently correlated with the evidence of cardiac dysfunction based on echocardiography (Table 5). There was a no stepwise increase in SIMD with increasing quartiles of admission and maximum hs-TnI (Table 6).

## 4. Discussion

In this study, we demonstrated that hs-TnI elevation was increased by up to 38% in septic shock patients, and of these, 65% patients showed cardiac dysfunction on an echocardiogram. The initial and peak hs-TnI levels showed a significant elevation in SIMD patients and were correlated with different types of dysfunctions. Peak hs-TnI (OR 1.03, 95%CI 1.01–1.06, *p* = 0.008) and ST-T wave changes in the electrocardiogram (OR 1.82, 95% CI 1.04–2.39) were associated with SIMD; however, the hs-TnI level at admission was not included in multivariate analysis.

Previous studies have evaluated the usefulness of different cardiac biomarkers in recognizing the early signs of myocardial injury without acute coronary syndrome [20,21]. Among these markers, a higher serum troponin level is a prognostic indicator of high mortality in sepsis cases. Its impact has had inconsistent reports because of differences in troponin types (troponin I or troponin T), disease severity, cutoff values, and the time to measurement [11,22,23,24]. Wilhelm et al. reported that non-survivors of septic patients had higher sensitivity troponin T on admission compared with survivors [22]. Similarly, a previous study from the United States showed the troponin T level on admission was associated with a higher in-hospital (OR 1.6; *p* = 0.003), and 1-year mortality (OR 1.3; *p* = 0.04) [24]. Another study reported that an elevated troponin-T, not initially, but on day 7 after admission, was associated with increased mortality (OR 1.38, *p* = 0.01) [23]. These studies tried to reveal correlations between troponin level and mortality. Mehta et al. reported that troponin I was an independent predictor of death and was correlated with lower LVEF (*p* < 0.001) [25], but, they included a relatively small number of patients (*n* = 16) and measured only LVEF via TTE.

Whether myocardial dysfunction induces troponin release remains a question of debate. Several studies correlated troponin levels and SIMD [25,26]. Røsjø and colleagues, evaluating hs-TnT in a large cohort of patients with sepsis, found that hs-TnT levels reflected cardiomyocyte injury but did not reliably identify SIMD [27]. Although there were different types of troponins, the pattern of the result is similar with the present finding that peak hs-TnI was associated with SIMD; however, hs-TnI level at admission was not. Furthermore, both admission and maximum hs-TnI did not demonstrate a stepwise increase of OR for SIMD. The underlying mechanism of troponin elevation in sepsis has not yet been elucidated. Contrary to coronary artery occlusion by atherosclerosis, it has been reported that coronary blood flows are similar or even increased in cases of sepsis [3]. A rapid incline and decline in the troponin level could be interpreted to mean that SIMD is a reversible myocardial ischemia [28]. Recently, some studies have found that nitric oxide, inflammatory cells, cytokines, complement, and mitochondrial dysfunction play complex roles in the pathways of cardiac dysfunction at both the cellular and molecular levels [29]. One of these effects is cell membrane permeability and functional alterations to ion channels, which contribute to the release of troponin from the intracellular space [30]. A delayed washout or an increasing level of troponin may correlate with ongoing myocardial injury. Because troponin can be quite readily and cost-effectively assayed in the clinic, repeated measurements may be a valid approach to identifying cardiac dysfunction.

Numerous echocardiographic parameters have been studied to date to help define SIMD [31,32,33]. According to the present findings, we evaluated the relationship between hs-TnI levels and the various types of SIMD. Initial hs-TnI was significantly higher only in patients with LV diastolic dysfunction. Bouhemad et al. also found that LV diastolic dysfunction is associated with troponin I elevation [31]. In addition, Landesberg et al. analyzed ICU patients with septic shock and reported that the troponin-T concentration correlated with LV diastolic dysfunction [32]. On the other hand, peak hs-TnI was significantly higher in the RV dysfunction group. Pulido et al. also reported that the serum troponin T level was higher in the RV dysfunction group; however, they had a different definition of RV dysfunction and the timing of troponin measurement [33].

Newly developed echocardiographic parameters, such as the LV longitudinal strain, ventricular arterial decoupling and speckle-tracking echocardiography, have shown better performance in predicting SIMD and mortality in septic shock patients than classical variables [34]. Landesberg et al. reported that the high-sensitivity troponin-T concentration correlated with LV diastolic dysfunction and RV dilatation, as measured by global strain, strain-rate imaging and 3D ventricular volume analysis [35]. Sanfilippo et al. reported that a lower e’ (standard mean difference 0.33; *p* = 0.02) and higher E/e’ (standard mean difference −0.33; *p* = 0.006), calculated using tissue Doppler imaging, are associated with mortality in septic patients [36]. A distinct advantage of the newly developed parameters is that they are independent or the volume status or vasopressors, but a limitation is that they are not as readily measured and applied as the standard parameters.

Our current study had several limitations of note. First, it was a retrospective cohort study of prospectively collected data from a single center only. Second, the times between the measurement of troponin and echocardiography exams were not consistent. Previous studies reported that patients’ cardiac function could be recovered fully to their premorbid state after 7–10 days. In our study, 35 (8.8%) patients were checked echocardiographically after 7 days. Among these, 21 (60%) showed no cardiac dysfunction and some of the normal findings could be the recovered state and could be influenced results. Third, our hospital cannot check cardiac troponin-T and there might be some difference among specific troponin types. However, a previous study reported that there was strong correlation between troponin-T and troponin-I [21]. Fourth, not all patients underwent baseline echocardiography and hs-TnI level testing prior to admission. Hence, we could not confirm the extent to which septic shock was responsible for the abnormalities based on a TTE.

## 5. Conclusions

About two-thirds of patients with an elevated hs-TnI level have various cardiac dysfunctions based on TTE. The peak troponin level is associated with SIMD based on TTE; however, the hs-TnI level at admission did not reliably identify SIMD. Serial measurements of troponin and EKG may be considered for patients with potential SIMD.

## Figures and Tables

**Table 1:** Baseline Characteristics of Patients with SIMD based on TTE.

Characteristics	Total *n* = 397	SIMD (−) Based on TTE *n* = 139	SIMD (+) Based on TTE *n* = 258	*p*-Value
Age	67.0 (58.0–75.0)	66.0 (54.0–75.0)	69.0 (60.0–76.0)	0.270
Male	224 (56.4)	83 (59.7)	141 (54.7)	0.342
Past illness				
HTN	131 (33.0)	37 (26.6)	94 (36.4)	0.057
DM	114 (28.8)	32 (23.0)	82 (31.9)	0.064
CAD	60 (15.1)	14 (10.1)	46 (17.8)	0.041 *
CKD	53 (13.4)	13 (9.4)	40 (15.5)	0.091
Pulmonary	58 (14.6)	21 (15.1)	37 (14.3)	0.882
Malignancy	130 (32.7)	50 (36.0)	80 (31.0)	0.370
Heart failure	25 (6.3)	5 (3.6)	20 (7.8)	0.130
Arrhythmia	46 (11.6)	14 (10.1)	32 (12.4)	0.517
EKG STTC	200 (50.4)	55 (39.6)	145 (56.2)	0.006 **
Vital signs				
Systolic BP	96.0 (79.0–121.0)	99.0 (80.0–123.5)	98.5 (79.0–127.0)	0.891
Diastolic BP	59.0 (49.0–76.0)	59.0 (49.0–73.0)	62.0 (48.8–80.0)	0.557
Heart rate	110.0 (90.0–128.0)	111.0 (91.5–125.0)	107.0 (89.0–128.0)	0.430
SOFA	9.0 (7.0–12.0)	9.0 (8.0–12.0)	9.0 (7.0–12.0)	0.427
Laboratory				
WBC (×10^3^/µL)	10.4 (4.2–16.9)	9.0 (3.9–16.9)	11.6 (5.1–17.0)	0.282
Hb (g/dL)	11.1 (9.2–13.1)	11.2 (9.1–13.5)	11.15 (9.2–13.1)	0.484
BUN (mg/dL)	29.0 (19.0–46.0)	31.0 (20.5–47.0)	29.0 (19.0–43.0)	0.463
Creatinine (mg/dL)	1.72 (1.10–2.97)	1.79 (1.11–3.16)	1.69 (1.08–3.01)	0.420
CRP (mg/dL)	13.9 (5.4–21.8)	15.7 (4.3–25.3)	13.3 (4.9–20.5)	0.433
Lactate (mmol/L)	4.1 (2.4–6.4)	4.2 (2.7–6.3)	3.9 (2.4–6.0)	0.424
Initial hs-TnI (ng/mL)	0.110 (0.044–0.318)	0.100 (0.040–0.258)	0.110 (0.050–0.480)	0.165
Peak hs-TnI (ng/mL)	0.539 (0.178–2.293)	0.390 (0.168–1.145)	1.535 (0.329–7.103)	<0.001 ***

Data are presented as a number (%) or median with interquartile ranges. * *p* < 0.05; ** *p* < 0.01; *** *p* < 0.001. Abbreviations: SIMD = sepsis-induced myocardial dysfunction; TTE = transthoracic echocardiography; HTN = hypertension; DM = diabetes mellitus; CAD = coronary artery disease; CKD = chronic kidney disease; EKG = electrocardiogram; STTC = ST segment and T wave change; BP = blood pressure; SOFA = sequential organ failure assessment; WBC = white blood cells; Hb = hemoglobin; BUN = blood urea nitrogen; CRP = c-reactive protein; hs-TnI = high sensitivity troponin-I.

**Table 2:** Type of Sepsis-induced myocardial dysfunction based on TTE.

Type of Cardiac Dysfunctions	Frequency (%)
LV dysfunction	221 (85.7%)
Systolic dysfunction	163 (63.2%)
Diastolic dysfunction	104 (40.3%)
RV dysfunction	97 (37.6%)
Wall motion abnormalities	186 (72.1%)

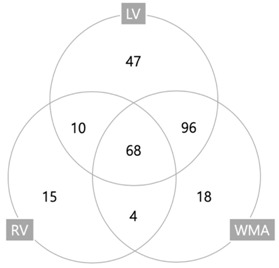

Data are presented as a number (%). Abbreviations: TTE = trans-thoracic echocardiography; LV = left ventricle; RV = right ventricle; WMA = wall motion abnormality.

**Table 3:** Echocardiographic Characteristics of Cohorts.

Parameter	Total *n* = 397	SIMD (−) on TTE *n* = 139	SIMD (+) on TTE *n* = 258	*p*-Value
LVIDs (mm)	33.0 (27.0–39.0)	29.0 (25.0–33.0)	35.0 (28.8–40.3)	<0.001 ***
LVIDd (mm)	48.0 (42.3–52.0)	45.5 (41.0–50.0)	59.0 (44.0–54.0)	<0.001 ***
LA (mm)	36.0 (32.0–41.0)	36.0 (31.0–38.8)	37.0 (33.8–41.0)	0.013 *
Aorta (mm)	34.0 (31.0–36.0)	34.0 (31.0–36.0)	34.0 (31.0–37.0)	0.566
LVPWs (mm)	13.0 (12.0–15.0)	14.0 (12.3–15.0)	13.0 (12.0–15.0)	<0.001 ***
LVPWd (mm)	9.0 (8.0–10.0)	9.0 (8.0–10.0)	9.0 (8.0–10.0)	0.116
ESV (mL)	42.0 (28.0–66.0)	31.5 (25.0–40.0)	49.5 (33.0–74.0)	<0.001 ***
EDV (mL)	90.0 (69.0–113.5)	81.5 (66.3–97.8)	98.5 (76.0–125.3)	<0.001 ***
IVSs (mm)	13.0 (11.0–14.0)	13.5 (12.0–15.0)	13.0 (11.0–14.0)	<0.001 ***
IVSd (mm)	9.0 (8.0–10.0)	9.0 (8.0–10.0)	9.0 (8.0–10.0)	0.173
LV mass (g)	148.6 (114.5–175.6)	144.8 (111.1–164.5)	159.0 (128.6–189.2)	0.029 *
LVEF (%)	55.0 (40.0–62.0)	61.0 (57.0–65.0)	49.0 (35.0–59.0)	<0.001 ***
Peak E velocity (cm/s)	69.0 (54.0–90.0)	62.0 (52.0–77.8)	69.0 (53.8–86.8)	0.090
Peak A velocity (cm/s)	77.0 (62.0–92.0)	75.0 (63.3–87.5)	75.0 (59.0–93.0)	0.111
Deceleration time (ms)	174.0 (140.0–213.0)	199.0 (155.3–234.3)	172.0 (139.5–207.5)	<0.001 ***
E/A ratio	0.84 (0.65–1.13)	0.87 (0.65–1.13)	0.86 (0.72–1.21)	0.707
E/e’ ratio	12.0 (9.0–15.0)	10.0 (9.0–12.0)	14.0 (10.0–17.0)	<0.001 ***

Data are presented as median with interquartile ranges. * *p* < 0.05; *** *p* < 0.001. Abbreviations: LVIDs = left ventricular internal diameter (systolic); LVIDd = left ventricular internal diameter (diastolic); LA = left atrium; LVPWs = left ventricle posterior wall (systolic); LVPWd = left ventricle posterior wall (diastolic); ESV = end-systolic volume; EDV = end-diastolic volume; IVSs = interventricular septum (systolic); IVSd = interventricular septum (diastolic); LVEF = left ventricle ejection fraction; E = peak early diastolic transmitral flow velocity; A = peak late diastolic transmitral flow velocity; e’ = peak early diastolic mitral annulus velocity.

**Table 4:** Cardiac biomarkers for predicting the various types of SIMD based on TTE.

SIMD	hs-TnI (ng/mL)	SIMD (−)	SIMD (+)	*p*
Any	Initial	0.100 (0.040–0.258)	0.110 (0.050–0.480)	0.165
	Peak	0.390 (0.168–1.145)	1.535 (0.329–7.103)	<0.001 ***
LV systolic	Initial	0.100 (0.040–0.323)	0.110 (0.050–0.527)	0.228
	Peak	0.540 (0.233–14.240)	1.609 (0.341–34.649)	<0.001 ***
LV diastolic	Initial	0.085 (0.312–0.289)	0.160 (0.050–0.513)	0.019 *
	Peak	0.739 (0.236–3.519)	0.903 (0.306–5.230)	0.177
RV	Initial	0.101 (0.040–0.305)	0.094 (0.050–0.574)	0.272
	Peak	0.546 (0.222–2.672)	2.478 (0.547–25.845)	<0.001 ***
WMA	Initial	0.100 (0.040–0.288)	0.110 (0.050–0.523)	0.869
	Peak	0.455 (0.205–1.680)	1.950 (0.374–9.741)	<0.001 ***

Data are presented as a number (%) or median with interquartile ranges. Number of any dysfunctions are 258, LV systolic dysfunctions are 163, LV diastolic dysfunctions are 104, RV dysfunctions are 186, and WMA are 186. * *p* < 0.05; *** *p* < 0.001. Abbreviations: SIMD = sepsis-induced myocardial dysfunction; TTE = transthoracic echocardiography; hs-TnI = high-sensitivity troponin-I; LV= left ventricle; RV = right ventricle; WMA = wall motion abnormalities.

**Table 5:** Multivariate logistic regression analysis of factors associated with SIMD based on TTE.

Variables	Univariate Analysis			Multivariate Analysis		
	OR	95% CI	*p*	OR	95% CI	*p*
CAD	1.595	0.820–3.105	0.169			
EKG STTC	1.689	1.115–2.561	0.013	1.822	1.210–2.744	0.004 **
Peak hs-TnI	1.032	1.008–1.057	0.008	1.033	1.009–1.057	0.008 **

** *p* < 0.01. Abbreviations: SIMD = sepsis-induced myocardial dysfunction; TTE = transthoracic echocardiography; OR = odds ratio; CI = confidence interval; CAD = coronary artery disease; EKG = electrocardiogram; STTC = ST segment and T wave change; hs-TnI = high-sensitivity troponin-I.

**Table 6:** Adjusted odds ratios of troponin level for SIMD.

Variables	Multivariate Analysis		
	OR	95% CI	*p*-Value
Initial hs-TnI			
Quartile 1	Reference		
Quartile 2	1.270	0.711–2.266	0.419
Quartile 3	0.805	0.443–1.462	0.477
Quartile 4	1.561	0.875–2.785	0.132
Maximum hs-TnI			
Quartile 1	Reference		
Quartile 2	1.241	0.671–2.295	0.491
Quartile 3	1.667	0.881–3.151	0.116
Quartile 4	5.496	2.843–10.624	0.001 ***

*** *p* < 0.001. Abbreviations: SIMD = sepsis-induced myocardial dysfunction; OR = odds ratio; CI = confidence interval; hs-TnI = high-sensitivity troponin-I.
